# Cerium Oxide/Graphene Oxide Hybrid: Synthesis, Characterization, and Evaluation of Anticancer Activity in a Breast Cancer Cell Line (MCF-7)

**DOI:** 10.3390/biomedicines11020531

**Published:** 2023-02-12

**Authors:** J. Saranya, P. Saminathan, Seshadri Reddy Ankireddy, Mohammed Rafi Shaik, Mujeeb Khan, Merajuddin Khan, Baji Shaik

**Affiliations:** 1 Department of Electronics and Communication Engineering, Rajalakshmi Engineering College, Chennai 602105, Tamil Nadu, India; 2 Sasaam Biologicals Lab Services, Ashok Nagar, Chennai 600083, Tamil Nadu, India; 3 Dr. Buddolla’s Institute of Life Sciences, Renigunta Road, Tirupati 517503, Andhra Pradesh, India; 4 Department of Chemistry, College of Science, King Saud University, P.O. Box 2455, Riyadh 11451, Saudi Arabia; 5 School of Chemical Engineering, Yeungnam University, Gyeongsan 38541, Republic of Korea

**Keywords:** CeO_2_/GO hybrid, Cis-CeO_2_/GO hybrid, MTT, FACS, AO/EB, MCF-7

## Abstract

In the present study, we used a simple ultrasonic approach to develop a Cerium oxide/Graphene oxide hybrid (CeO_2_/GO hybrid) nanocomposite system. Particle size analysis, Fourier Transform Infrared Spectroscopy (FTIR), Scanning Electron Microscopy (SEM), and X-ray Diffraction (XRD) have been used to analyze the physio-chemical characteristics of the developed nanocomposite. The synthesized hybrid system has also been examined to assess its anticancer capability against MCF-7 cell lines and normal cell lines at different sample concentrations, pH values, and incubation intervals using an antiproliferative assay test. The test results demonstrate that as sample concentration rises, the apoptotic behavior of the CeO_2_/GO hybrid in the MCF-7 cell line also rises. The IC_50_ was 62.5 µg/mL after 72 h of incubation. Cytotoxicity of cisplatin bound CeO_2_/GO hybrid was also tested in MCF-7 cell lines. To identify apoptosis-associated alterations of cell membranes during the process of apoptosis, a dual acridine orange/ethidium bromide (AO/EB) fluorescence staining was carried out at three specified doses (i.e., 1000 µg/mL, 250 µg/mL, and 62.5 µg/mL of CeO_2_/GO hybrid). The color variations from both live (green) and dead (red) cells were examined using fluorescence microscopy under in vitro conditions. The quantitative analysis was performed using flow cytometry to identify the cell cycle at which the maximum number of MCF-7 cells had been destroyed as a result of interaction with the developed CeO_2_/GO hybrid (FACS study). According to the results of the FACS investigation, the majority of cancer cells were inhibited at the R3 (G2/M) phase. Therefore, the CeO_2_/GO hybrid has successfully showed enhanced anticancer efficacy against the MCF-7 cell line at the IC50 concentration. According to the current study, the CeO_2_/GO platform can be used as a therapeutic platform for breast cancer. The synergetic effects of the developed CeO_2_/GO hybrid with the MCF-7 cell line are presented.

## 1. Introduction

As per the cancer statistics revealed by ICMR in the year 2021, India has over 200,000 new cases on average and 43,000 deaths due to breast and cervix cancer. Approximately 80% of the woman from the age group of 45 years and 43% of the woman from age group of 65 years and above were diagnosed with breast cancer. Despite the fact that it can destroy and impair organs, cancer is an aberrant or uncontrolled cellular proliferation that can invade other healthy tissues of the body. Conversely, one of the leading causes of death for women in wealthy countries today is breast cancer. Chemotherapy treatment may last for hours, days, weeks, or even months, depending on how far radiotherapy and chemotherapy for cancer treatment have come. Radiation therapy sometimes causes tissue damage near organs [1]. Both of these treatments carry risks, are expensive, and have specific side effects. As a result, cancer requires a straightforward and effective treatment. Much emphasis has been paid to the application of nanoscience in the treatment of cancer [2,3].

Nano-sized materials, which are typically 1–100 nm, are used in medical nanotechnology. These materials are used in the design and production of medicinal medications and devices [4]. One of the main advantages of nanomaterial-based cancer therapy over free medicines is targeted delivery. Targeted delivery based on nanoparticles has recently advanced. Using either passive or active targeting, the concept of targeted delivery seeks to precisely target particular cancer cells [5]. Nanomaterials are widely used in cancer radiation therapy, immunotherapy, and combined therapy for advancing the therapy strategy. Currently, inorganic nanoparticle-based nanomaterials are increasingly being explored for a variety of medicinal applications, including as anticancer agents [6]. Among various inorganic nanoparticles, metal oxides are more favored for biomedical applications, including drug delivery, cell imaging, and cancer treatment [7]. Metallic and/or metal oxide nanoparticles demonstrate characteristic physicochemical properties including excellent optical, magnetic, and electrical activities, and so on [8]. Out of several metal oxides, iron, nickel, cerium cobalt-based oxide nanoparticles have been considerably used for several biomedical applications due to their superior optical and magnetic properties. These materials have been known to exhibit effective biological potential, such as the ability to induce DNA damage, oxidative stress, genotoxic effects, and anti-inflammatory responses. Due to these effects of metal oxide NPs, they are considered as potential therapeutic anticancer agents. In these regards, various magnetic metal and metal oxide nanoparticle (MNP) systems, have been successfully utilized in cancer treatment, which have shown significant advancements in a number of pre-clinical cancer theranostics applications [9]. 

For example, to diminish the excessive usage of aggressive anticancer drugs, different options are required, including the application of copper oxide (CuO) NPs, as reported by Dringen et al., who have explored the effect of uptake and toxicity of ionic copper by C6 glioma cells [10]. Besides, many other studies have suggested that a variety of magnetic NPs induce selective autophagy in cancerous cells without affecting normal cells, and thereby exhibit an intrinsic toxicity specifically toward cancer cells, which would enhance their therapeutic effect [11]. MNPs can display toxicity through mechanisms such as the production of reactive oxygen species (ROS), generation of ROS directly from the NP surface, and through alteration of mitochondria [12]. CeO_2_ NPs receive a lot of attention in nanoscience because they are useful as catalysts, fuel cells, and antioxidants in biosystems [13]. In general, cerium can exist in both Ce^3+^ and Ce^4+^ oxidation states. The surface Ce^3+^: Ce^4+^ ratio is influenced by the microenvironment. As a result, the microenvironment and synthesis process are key variables in the estimate of CeO_2_NPs’ biological activity [14]. CeO_2_NPs have reportedly recently gained popularity as attractive materials in a number of significant domains, including analysis, drug delivery, and biomedicine [15,16,17,18,19]. On the other hand, Carbon atoms are arranged in single layers in the two-dimensional compound called as graphene. Due to its unique physical, chemical, and mechanical properties, nano-sized graphene and its associated compounds have attracted a great deal of interest for usage in various biological applications [20,21]. Because of its high surface to volume ratio, outstanding loading capacity, and good near-infrared (NIR) absorbance, one prominent graphene derivative, graphene oxide (GO), has been widely used in biosensors, drug and gene delivery vectors, imaging contrast agents, and photothermal therapy mediators [22,23,24,25,26,27]. 

In addition, nanomaterials are becoming popular as drug vehicles to achieve fast-cytoplasmic drug release to cancer cells. Particularly, the lysosomal pH-triggered fast drug release nanoparticles have gained considerable attention. Since, due to their excellent physicochemical properties, including high stability and smaller size, nanomaterials, can be easily internalized through endocytosis and end up in endosomes and lysosomes, where the pHs is approximately 5.5 (endosomes) and 5 (lysosomes) [28]. In this regard, Shen et al. have prepared cisplatin-loaded nanoparticles with pH-responsive polymeric cores, which dissolved at pH < 6 and are rapidly internalized and transferred to lysosomes [29]. Similarly, Gao et al. have reported that doxorubicin conjugated magnetite nanoparticles with an effective pH response have demonstrated much higher anticancer activity than free DOX [30]. In addition to pH, particle size and surface composition also influence anticancer properties, such as cellular uptake in vitro and whole-body transport in vivo [31]. 

According to the characterization of these cell lines, cancer cell lines are an excellent model for research into the molecular mechanisms that cause the disease. The use of cancer cell lines for research has been widespread, and they have proven to be an effective tool for the genetic approach [32]. A deeper knowledge of the unregulated genes was made possible by the use of cancer cell lines. Owing to these changes, it has been confirmed that the CeO_2_ NPs were fabricated on the surface of GO NPs. Experiments in cell lines (in vitro tests) are not alternatives to animal tumor models (in vivo experiments). Experiments in cell lines are just one step in the preclinical investigation of the potential new drug, after which, in vivo experiments are performed in tumor bearing mice. To examine genetic, epigenetic, and cellular pathways; analyze proliferative dysregulation, apoptosis, and cancer development; establish important molecular markers; and screen and characterize cancer treatments, the appropriate in vitro model must be employed in cancer research [33]. Using an anti-proliferative assay, this work seeks to synthesize a CeO_2_/GO nanocomposite and evaluate its anticancer potential in MCF-7 cell lines and normal cell lines. To further support the use of the developed nanoplatform as a drug delivery platform, CeO_2_/GO nanocomposites were used as an anticancer agent in therapeutics.

## 2. Results and Discussion

### 2.1. XRD Analysis

**Figure 1:**
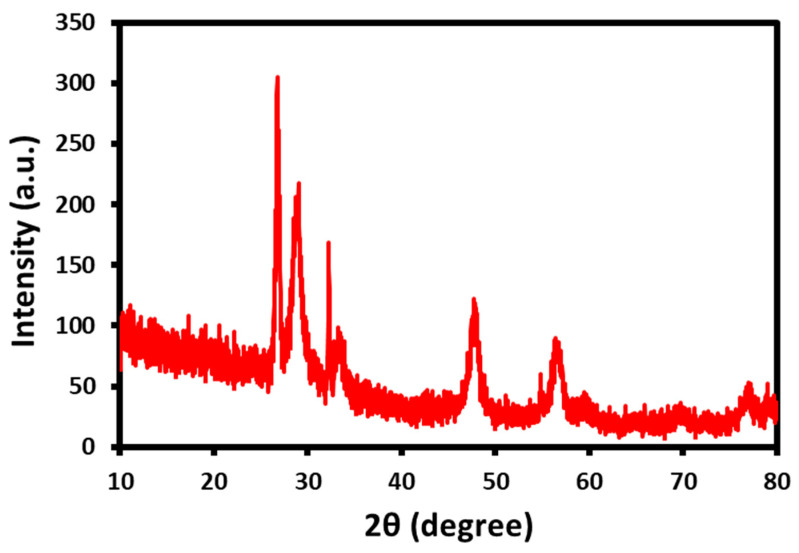
X-ray diffraction patterns of the CeO_2_/GO nanocomposite.

The phase purity and nanocomposite crystallinity were investigated using the powder XRD analysis, as depicted in Figure 1. The results show that considerable diffraction peaks were identified 2θ at 26.4°, 29.7°, 47.3°, 57.8°, and 78.1° for the presence of the lattice planes of (111), (200), (220), (222), and (333), respectively. However, there was a small peak observed with a less intense 2θ at 19.2°, which was ascertained for the presence of a graphitic core of the GO. The degree of crystallinity and peak intensity both deteriorate when CeO_2_ is deposited on the surface of GO sheets. Based on these observations, the GO platform has been used for the fabrication of the CeO_2_ NPs on their surface.

### 2.2. UV-Visible Analysis

**Figure 2:**
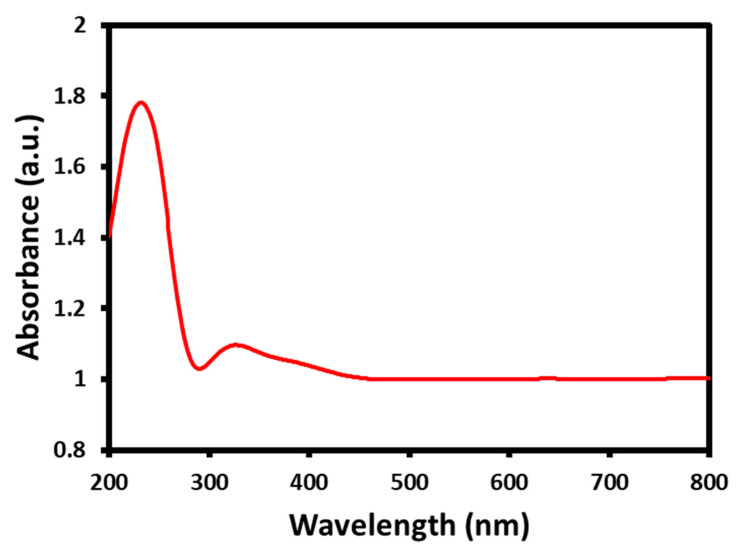
UV-Visible analysis of the CeO_2_/GO nanocomposite.

Initially, the authorization of the development of the GO/CeO_2_ nanocomposite was examined using UV–Visible analysis (Figure 2). The UV-Vis results exhibited the absorption band of an intense surface plasmon resonance (SPR) absorption peak at 235 nm, which corresponds to π—π* transitions of the remaining sp^2^ C=C bonds [34,35], and the another absorption peak around 300–350 nm, which corresponds to the plasma resonance of CeO_2_ [36]. The ultraviolet–visible analysis clearly indicates the formation of the GO/CeO_2_ nanocomposite.

### 2.3. Surface Morphology Analysis

**Figure 3:**
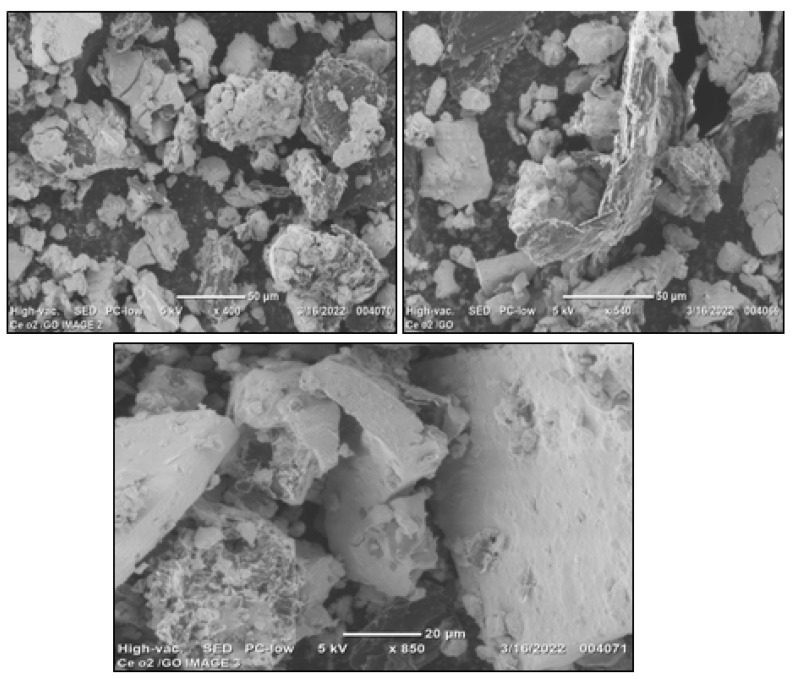
SEM images of the CeO_2_/GO nanocomposite with a magnification of 400×, 540×, and 850×.

An outstanding possibility for morphological analysis and nanocomposite size analysis is provided by SEM inspection. The typical SEM micrograph of the synthesized CeO_2_/GO nanocomposite surface morphology shows irregular vesicles at the range of 50 µm at 400× magnification and 540× magnification, respectively. Further, at 850× magnification, cubic irregular vesicles at the size of 20 µm with detailed encapsulation of CeO_2_/GO NPs were found, as shown in Figure 3. Previous studies have shown that nanoparticles without any agglomeration exhibit stable behavior because they have surface charges [37]. The hierarchical structure of the CeO_2_/GO nanocomposite improves anticancer activity, and the same is affirmed through in vitro studies using MCF-7 cell lines and normal HeLa cells.

### 2.4. FT-IR Analysis

**Figure 4:**
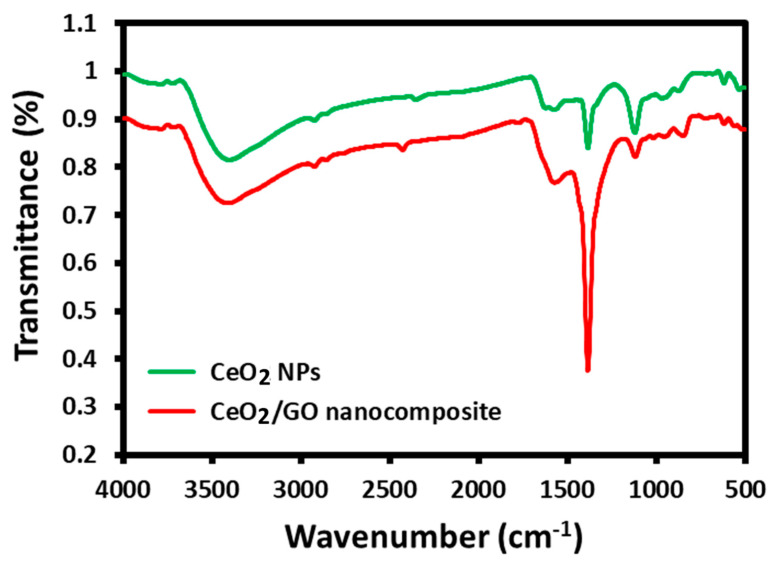
FT-IR analysis of CeO_2_ NPs and Ce_2_O/GO nanocomposites.

The functional group changes of CeO_2_ NPs before and after treatment with GO were investigated using the FT-IR analysis, as presented in Figure 4. According to the image, several characteristic absorption peaks were observed at 3401, 1580, 1379, and 1125 cm^−1^, which are responsible for the presence of stretching frequencies of hydroxyl, Ce-OH, bending frequencies of Ce-O, and metal oxide bonds, respectively. However, significant changes were observed in the aforementioned peaks due to the incorporation of GO. The hydroxyl and Ce-OH bending peaks were shifted to higher and lower wavenumbers, respectively. Interestingly, the bending frequency of Ce-O has increased dramatically due to the presence of GO immobilization. Owing to these changes, it has been confirmed that the CeO_2_ NPs were fabricated on the surface of CeO_2_/GO NPs.

### 2.5. Study of Cytotoxicity Effects of CeO_2_/GO NC and Cis-CeO_2_/GO NC in the MCF-7 Cell Line and Normal Cell Line Using the MTT Assay

**Figure 5:**
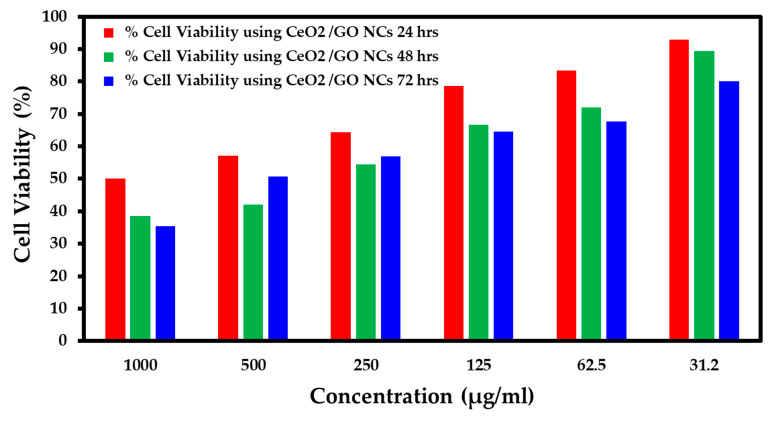
Percent cell viability for the normal cell line (pH 7.2).

**Figure 6:**
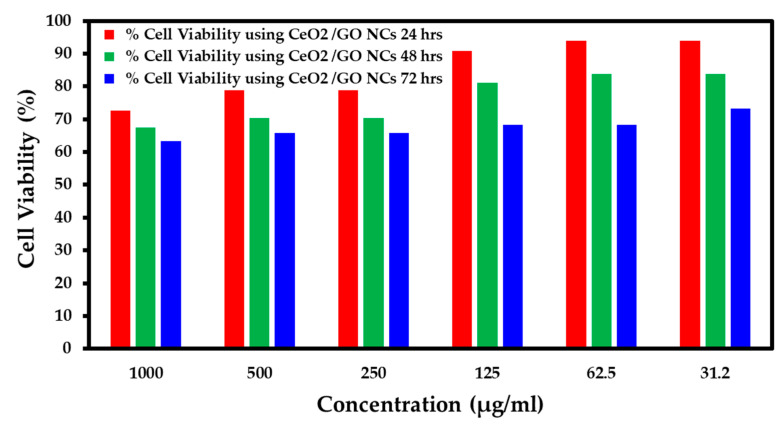
Percent cell viability for the normal cell line (pH 6).

**Figure 7:**
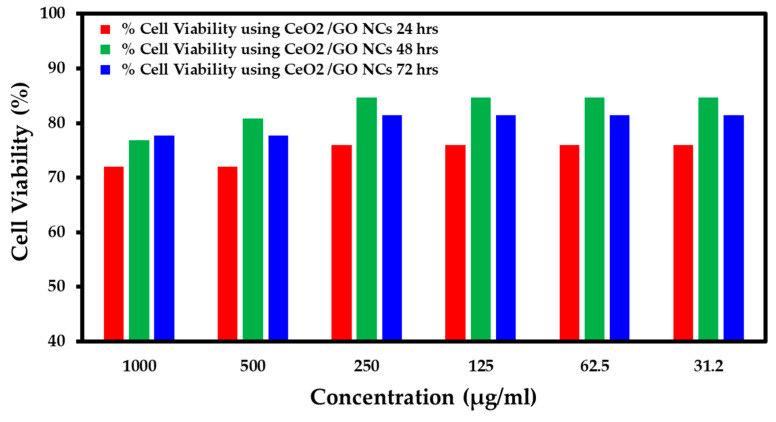
Percent cell viability for the normal cell line (pH 8.5).

**Figure 8:**
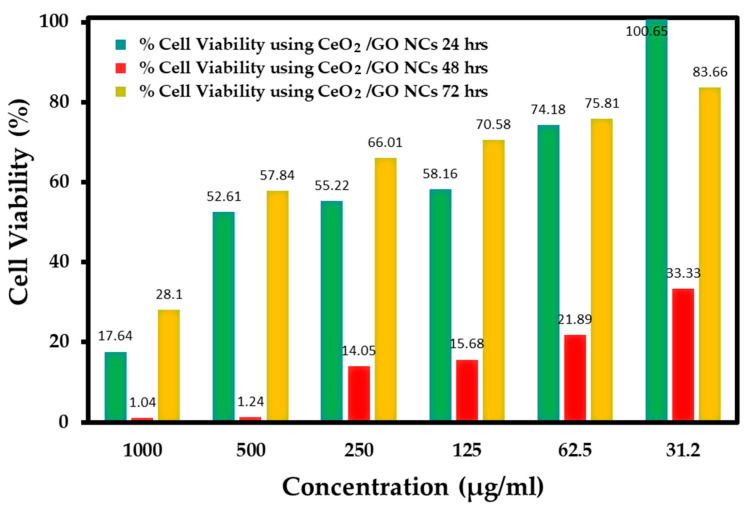
Percent cell viability of CeO_2_/GO hybrid in the MCF-7 cell line without drug.

**Figure 9:**
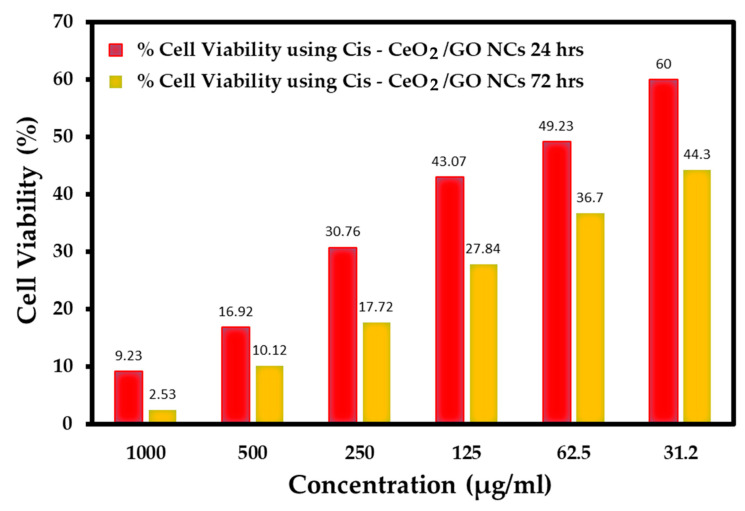
Percent cell viability of Cis-CeO_2_/GO hybrid in the MCF-7 cell line.

The cytotoxicity effects of the developed CeO_2_/GO nanocomposite upon interaction at various concentrations, such as 1000 µg/mL, 500 µg/mL, 250 µg/mL, 125 µg/mL, 62.5 µg/mL, and 31.2 µg/mL, with nearing 100,000 normal cells under different pH values, such as 7.2, 6, and 8.5, are presented in Figure 5, Figure 6 and Figure 7, respectively. Further, the interaction of the CeO_2_/GO nanocomposite with the MCF-7 cell line with and without cisplatin drug is presented in Figure 8 and Figure 9.

During the interaction of 1000 µg/mL of CeO_2_/GO with a normal cell line under pH 7.2, the % live cells was 50, under pH 6, the % live cells was 72.72, and for pH 8.5, the % live cells was 72. Similarly, when the minimum concentration of 31.2 µg/mL of the CeO_2_/GO nanocomposite was combined with a normal cell line under pH 7.2, the % live cells was 92.85, under pH 6, the % live cells was 93.93, and for pH 8.5, the % live cells was 76. Based on the observation, the ideal pH condition for which the developed CeO_2_/GO nanocomposite synergized well with a normal cell line and showed minimal toxicity effects (more live cells are seen) is pH 6, as shown in Figure 5, Figure 6 and Figure 7.

The cytotoxicity effects of the developed CeO_2_/GO NCs were recorded against MCF-7 cells, as shown in Figure 8. At the maximum incubation period of 72 h, when the maximum concentration of 1000 µg/mL interacted with MCF-7 cells, the % live cells recorded was 28, and at the 31.2 µg/mL concentration, the % live cells recorded was 83.66. These observations further affirm the anticancer effect of the developed CeO_2_/GO nanocomposite. Further, the interaction of Cis-CeO_2_/GO NCs with the MCF-7 cell line was carried out and observations were recorded at 24 and 72 h, as shown in Figure 9. For 1000 µg/mL of Cis-CeO_2_/GO nanocomposite, the % live cells was 2.53, and for 31.2 µg/mL, the % live cells was 44.3. A lattice of oxygen surrounds the cerium center of cerium oxide nanoparticles (NPs) [38]. Under neutral pH conditions, these NPs act as antioxidants and cytoprotectants in healthy cells. Additionally, in an acidic solution, these NPs have prooxidant and cytotoxic effects, which is a feature of tumor cells. The anticancer effects of cerium oxide NPs were investigated in vitro, and the findings indicated that cerium oxide NPs suppress tumor cell proliferation [39]. Another study revealed that cerium oxide NPs with (10 µg/mL) can inhibit gastric tumor cell proliferation and reduce tumor cell migration [40]. Due to its high surface to volume ratio and the unique physicochemical characteristics of CeO_2_ NPs and GO, the synthesized CeO_2_/GO nanocomposite exhibit outstanding dose-dependent anticancer activity through DNA damage and the generation of reactive oxygen species (ROS). Further, Figure 9 demonstrates the increased cytotoxicity effects of Cis-CeO_2/_GO NPs when added with cisplatin on the MCF-7 cell line. This affirms the effective binding of anticancer drug molecules with the CeO_2/_GO hybrid.

### 2.6. Direct Fluorescence Microscopic Analysis of Apoptosis Induction

**Figure 10:**
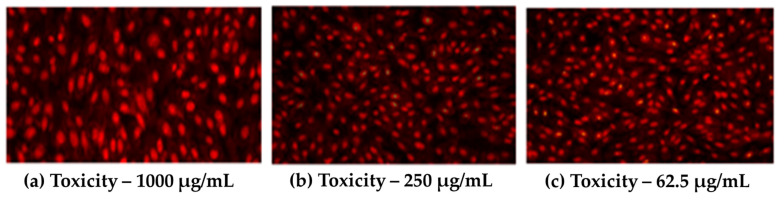
Microscopic fluorescent images of (**a**) MCF-7 cells with 1000 µg/mLCeO_2_/GO hybrid, (**b**,**c**) MCF-7 cells with 250 µg/mL and 62.5 µg/mL concentrations of CeO_2_/GO hybrid complexes, respectively.

An acridine orange/ethidium bromide (AO/EB) dual stain study was performed to detect the presence of live and dead cells using fluorescence emission under an in vitro microenvironment. The cells were fixed in a 3:1 ratio of ethanol and phosphate buffer solution (PBS) for 1 h at room temperature. The cells treated with the CeO_2_/GO hybrid nanocomposite were labeled with a 1:1 ratio of AO and EB in PBS and were incubated. Then, the excess unbinding dye was removed by washing with PBS and the stained cells were visualized using a fluorescence microscope, as shown in Figure 10.

To identify the presence of live and dead cells using fluorescence emission in an in vitro microenvironment, a dual stain analysis employing acridine orange/ethidium bromide (AO/EB) dyes were conducted. For 1 h at room temperature, the cells were fixed in a 3:1 mixture of ethanol and phosphate buffer solution (PBS). After being treated with the CeO_2_/GO hybrid nanocomposite, the cells were incubated with AO and EB in a 1:1 ratio in PBS. Following a PBS wash to remove any excess unbinding dye, stained cells were viewed under a fluorescence microscope, as shown in Figure 10.

These pictures showcase the morphological alterations and color emission variations that result from mixing acridine orange (AO) and ethidium bromide (EB) dyes in an in vitro environment. More dead cells are shown in Figure 10a, and they appear red because they can absorb the ethidium bromide (EB) dye by itself. This affirms the apoptotic behavior of the developed CeO_2_/GO NCs. Figure 10b,c shows the coexistence of living and dead cells as a result of the live cells’ uptake of the acridine orange (AO) dye and the dead cells’ uptake of the ethidium bromide (EB) dye. This study demonstrates the morphological changes that cell lines experienced when they interact with different CeO_2_/GO NC concentrations.

### 2.7. Cell Cycle Analysis

**Figure 11:**
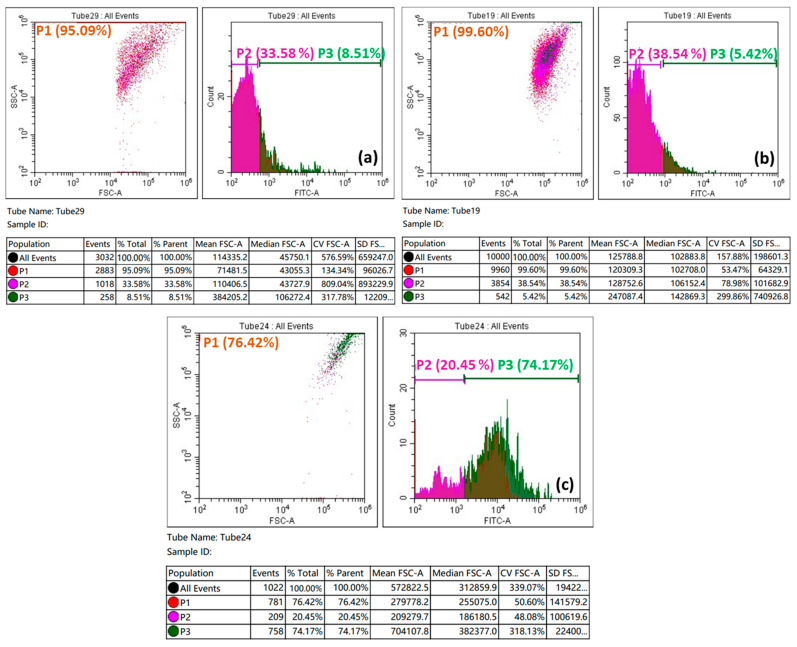
Flowcytometry of (**a**) untreated MCF-7 cells, and (**b**,**c**) treated MCF-7 cells with 62.5 µg/mL and 31.2 µg/mL concentrations of CeO_2_/GO hybrid complexes.

This study aims to determine the phase cycle at which the majority of cancer cells were destroyed. Figure 11 shows the apoptotic behavior of a produced CeO_2_/GO hybrid at two different concentrations (62.5 µg/mL and 31.2 µg/mL).

Figure 11a represents untreated MCF-7 cells with a total of 3032 cells, which were subject to cell cycle analysis. In phase 1, almost 5% of cancerous cells were dead, in phase 2, 66% of cancerous cells were dead, and in phase 3, 91.49% of cells were dead. The cell death recorded here is an outcome of the apoptotic behavior of cancer cells. Figure 11b represents recordings from cell cycle analysis of a total of 10,000 MCF-7 cells, which were synergized with 62.5 µg/mL of CeO_2_/GO NCs. Here, the second cell cycle, which only contains 3854 living cells, records the highest levels of apoptotic behavior. Figure 11c represents treated HeLa cells with a total of 1022 cells, which were subject to cell cycle analysis. Upon interaction of MCF-7 cells with 31.2 µg/mL, maximum cell cycle arrest was found in cell cycle 2. Cell death rate is more effective in phase 2 rather in the rest of the phases, and the same further confirms the anticancer properties of the developed CeO_2_/GO nanoplatform.

## 3. Materials and Methods

### 3.1. Materials

All chemicals and solvents utilized in this inquiry were of the highest purity and quality for analysis. Sodium hydroxide (NaOH) and cerous nitrate hexahydrate (CeN_3_O_9_·6H_2_O) were bought from Fischer Scientific, Mumbai, India. Utilizing the RIGAKU micro flux 2C, Tokyo, Japan, the X-ray Diffraction (XRD) spectrum of the CeO_2_/GO hybrid nanosystem was captured. A PerkinElmer lambda 35 (Waltham, MA, USA) UV/Vis spectrophotometer was used for the optical measurements. Using Scanning Electron Microscopy (SEM) produced by TESPON, Tokyo, Japan, surface morphology was determined. Particle size analysis was done using the Horiba-Particle Analyzer. A spectrophotometer was used to capture the absorption spectra (Labman scientific instruments, Chennai, India). Confocal images of HeLa cells were obtained using a Metzer Inverted confocal microscope, Mumbai, India.

### 3.2. Preparation of Cerium Oxide Nanoparticles (CeO_2_NPs)

A cost-effective wet chemical method was used to synthesize cerium oxide nanoparticles (CeO_2_ NPs) at room temperature. The first step was to dissolve 0.1 M of cerous nitrate hexahydrate (CeN_3_O_9_·6H_2_O) in 100 mL of pure water. The resulting 100 mL cerous nitrate hexa hydrate solution was then mixed with 50 mL of 0.5 M sodium hydroxide (NaOH) solution. The resultant solution was kept under constant stirring for 2 h and color change was detected. Further stirring was done until a yellowish solution appeared, confirming the presence of CeO_2_ NPs. This yellowish solution was set aside to allow the CeO_2_ nanoparticles to settle before being rinsed three times with distilled water. It was then annealed at 350 °C and dried at 150 °C. The powder that was so obtained was put to use for other analytical tasks.

### 3.3. Preparation of Graphene Oxide (GO)

Graphite powder (2 g) was added to concentrated H_2_SO_4_ (46 mL) at 0 °C in an ice bath and stirred for 5 min. NaNO_3_ (1 g) was then added and the solution was stirred for 5 min. KMnO_4_(6 g) was added slowly into the resulting mixture and stirring continued for 30 min at 35 °C. H_2_O (96 mL) was then added dropwise to the above mixture and the temperature was increased to 98 °C and maintained for 30 min; then, a further aliquot of H_2_O (280 mL) was added and stirring was continued for 10 min. Finally, aqueous H_2_O_2_ (30 mL, 30%) was added dropwise [37]. 

### 3.4. Preparation of CeO_2_/GO Nanocomposite

We used 100 mL of distilled water to dilute 1 g of prepared CeO_2_ NPs powder before it was ultrasonically processed for 30 min. Following that, 0.25 g of graphene oxide (GO) was diluted in 50 mL of distilled water and subjected to 2 h of ultrasonication. To create a nanocomposite solution, the two solutions containing the mixture are finally blended. Additionally, the combinations underwent two hours of stirring after being combined in order to produce a homogenous solution. After allowing the materials to settle, they were three times washed with distilled water to produce CeO_2_/GO NCs.

### 3.5. Preparation of Cis-CeO_2_/GO Hybrid Nanocomposite

At first, 1000 µg/mL of cisplatin were added to a well containing HeLa cell line. Then for each of the eight concentrations (7.8, 15.6, 31.2, 62.5, 125, 250, 500, and 1000 µg/mL) half of the concentration will be (CeO_2_/GO) sample and rest half will be cisplatin (for instance, 500 µg/mL of cisplatin and 500 µg/mL of CeO_2_/GO are present in a 1000 µg/mL stock solution). The same steps are repeated for additional concentrations. The Cis-CeO_2_/GO hybrid was developed using the aforementioned process, and it was then subject to an MTT assay routine to estimate the % cell viability.

### 3.6. Cytotoxicity Studies

#### 3.6.1. Cell Lines

The Veterinary College in Vepery, Chennai provided the MCF-7 cell line. The cells were kept alive at 37 °C in a humidified atmosphere of 50 μg/mL CO_2_ in Minimal Essential Medium (MEM) supplemented with 10% FBS, penicillin (100 U/mL), and streptomycin (100 μg/mL).

#### 3.6.2. Reagents

MEM was acquired from Hi Media Laboratories, Fetal Bovine Serum (FBS) from Cistron Laboratories, Coimbatore, Tamil Nadu, Inida and Trypsin, Methylthiazolyldiphenyl-tetrazoliumbromide (MTT), and Dimethyl Sulfoxide (DMSO) from Sisco Research Laboratory Chemicals, Mumbai, India. Other chemicals and reagents were obtained from Sigma-Aldrich, Mumbai, India.

#### 3.6.3. Evaluation of the Anticancer Activity of the CeO_2_/GO Hybrid and Cis-CeO_2_/GO Hybrid

The procedure incorporated for evaluation of the CeO_2_/GO hybrid and Cis-CeO_2_/GO hybrid are presented in supplementary sections. The UV Spectrophotometer was used to measure the absorbance at 570 nm. The following formula was used to determine the percentage of viable cells:

% cell viability = Optical density of treated cells/Optical density of control cells × 100

Each assay includes a cell control and sample control to provide a comprehensive evaluation of cell viability and the same were plotted as graphs by taking the concentration of the sample on the X-axis and the % cell viability on the Y-axis. 

### 3.7. Dual Staining (Acridine Orange and Ethidium Bromide Staining)

Acridine orange can stain both living and dead cells. Ethidium bromide only stains cells that have lost the integrity of their membranes. Cells that are alive will be green. As a result of chromatin condensation and nuclear fragmentation, early apoptotic cells will stain green and have brilliant green spots in their nuclei. In addition to incorporating ethidium bromide and staining orange, late apoptotic cells also exhibit contracted and typically broken nuclei in contrast to necrotic cells.

### 3.8. Flow Cytometry Study

Cell cycle arrest was investigated using a protocol [29], and the experimental procedure is provided under the supplementary section. The flow cytometer was used to examine the cell phase distribution in the labeled cells.

## 4. Conclusions

In conclusion, a CeO_2_/GO hybrid nanocomposite has been developed using an easy and affordable ultrasonic technique. The physio-chemical characteristics were examined using analysis tools such as XRD, Particle Size Analysis, SEM, and FTIR. Scanning electron microscopy was utilized to confirm the surface morphology (SEM). The particle size of the developed CeO_2_/GO hybrid nanocomposite was found to be 68.94 µm on average, which results in a high surface to volume ratio and improved anticancer efficacy in the MCF-7 cell line. The obtained CeO_2_/GO hybrid was subject to qualitative analysis, such as the MTT assay and dual stain study. The outcome of this study resulted in the identification of effective anticancer properties at the 62.5 µg/mL CeO_2_/GO hybrid concentration. To determine the cell cycle phase at which the majority of cancer cells were arrested, quantitative analysis (FACS) was performed on three sets of CeO_2_/GO concentrations, including the IC_50_ concentration. In comparison with the Cis-CeO_2_/GO hybrid, the CeO_2_/GO hybrid is superior. The reason behind is that at t = 24 h and at IC_50_ 62.5 µg/mL, the % cell viability is 21.89 for the CeO_2_/GO hybrid and 36.7 for the Cis-CeO_2_/GO hybrid. Maximum % cell inhibition is recorded for the CeO_2_/GO hybrid. This work primarily focuses on the assessment of the cytotoxic behavior of the CeO_2_/GO hybrid on MCF-7 cells under in vitro conditions. Based on the results from in vitro studies, the prepared CeO_2_/GO hybrid can be used as a theranostic platform for breast cancer. Thus, for future work, the developed CeO_2_/GO hybrid can be a potential subject for in vivo analysis and clinical trials for further confirmation and proceedings. 

## Data Availability

Data contained within the article.
